# Analysis of anthocyanins and total flavonoids content in functional rice and its recombination inbred lines

**DOI:** 10.3389/fpls.2023.1113618

**Published:** 2023-03-15

**Authors:** Xinyi Zhou, Wei Xie, Hao Jing, Junru Fu, Maomao Li, Jianmin Bian, Jie Xu, Yicong Cai, Haohua He, Dahu Zhou

**Affiliations:** ^1^ Key Laboratory of Crop Physiology, Ecology and Genetic Breeding, Ministry of Education, College of Agronomy, Jiangxi Agricultural University, Nanchang, China; ^2^ National Engineering Research Center of Rice (Nanchang), Key Laboratory of Rice Physiology and Genetics of Jiangxi Province, Rice Research Institute, Jiangxi Academy of Agriculture Sciences, Nanchang, China

**Keywords:** functional rice, ZBXN 1, flavonoids, anthocyanins, recombinant inbred lines (RILs)

## Abstract

Anthocyanin is one of the flavonoids, which has strong antioxidant properties. Functional rice rich in anthocyanins can not only improve immunity, but also anti-radiation, beauty, anti-aging effect, very popular in the market. In this study, we used Zibaoxiangnuo 1 (ZBXN 1), a functional rice variety which is rich in total flavonoids and anthocyanins, as the experimental material to construct Recombination Inbred Lines (RILs) with Minghui63 (MH63), a variety without anthocyanins. The contents of anthocyanins and total flavonoids of RILs and two parents were determined for three consecutive generations. The average anthocyanin content of parent ZBXN 1 was 319.31 mg/kg, and the anthocyanin inheritance of RIL population was relatively stable, with 10 samples higher than ZBXN 1. In addition, there was no significant difference in the total flavonoids content between the two parents, the total flavonoids content of Z25 in RIL population was 0.33%. Based on these studies, we believe that ZBXN 1 has abundant and stable anthocyanins, which can be used as an intermediate breeding material for breeding high-quality varieties with high anthocyanins, and lay a foundation for breeding more anthocyanin-rich rice varieties.

## 1 Introduction

With the growing standard of living, the market isn’t content with the yield of rice any more, but it more cares about its quality and nutritional value. Functional rice is a special rice variety with rich nutrients. The efficacies and effects of total flavonoids include preventing cardiovascular system diseases, having anti-oxidative, antibacterial, anti-inflammatory, and antitumour, and participating in hormone regulation and liver protection (Jiang et al., 2016). Anthocyanins are also kinds of flavonoids. The medical nutrition industry believes that anthocyanins contain strong antioxidant properties, which can not only improve immunity but also can resist radiation, beauty and delay ageing effect (Yousuf et al., 2016).

Anthocyanin is one of the flavonoids, which widely exists in plants. Anthocyanin can help plants resist pathogens, which is related to the stress resistance of plants (Lorenc-Kukula et al., 2005). In addition, anthocyanin has strong antioxidant activity, and has the health care functions of inhibiting inflammation and resisting cancer (Seeram et al., 2001; Cvorovic et al., 2010). Anthocyanins are formed from phenylalanine in plants through three stages and multi-step reactions (Zhang et al., 2014). In the first stage, phenylalanine was catalyzed by phenylalanine ammonia-lyase (PAL), Cinnamate-4-hydroxylase (C4H) and 4-coumarate:CoA ligase (4CL) to synthesize 4-coumarate:CoA. This stage is the common pathway of anthocyanin and flavonoid metabolism synthesis. Then in the second stage, 4-coumarate:CoA was catalyzed by chalcone synthase (CHS) to form chalcone, then Chalcone was catalyzed by Chalcone isomerase (CHI) to form naringenin, and naringin was catalyzed by Flavanone 3-hydroxylase (F3H) to finally synthesize flavanonol. This is the most critical period for the synthesis of flavonoid substances such as anthocyanin, and it is the branch of anthocyanin synthesis and biological total flavonoids. In the last stage, flavanonol was reduced by dihydroflavonol 4-reductase (DFR) to form colorless anthocyanins, which were catalyzed by anthocyanin synthase (ANS/LDOX) to form different kinds of anthocyanins. Finally through a series of complex methylation, acylation, hydroxylation, glycosylation derivative modification to form different colors and categories of anthocyanins (Holton and Cornish, 1995; Mol et al., 1998; Dubos et al., 2008; Hong et al., 2012). They are influenced by many regulatory genes. The regulation of anthocyanin metabolic pathways occurs mainly at the transcriptional level of structural genes. Its expression is stimulated or repressed through the association of one or more transcription factors and the corresponding cis-elements on the promoters of structural genes (Nicola A. Ramsay, 2005). It was found that MYB-R2R3-type, basic helix-loop-helix [bHLH]-type and WD40-type repeat proteins, these three types of transcription factors, can regulate the anthocyanin metabolic pathway (Sakamoto et al., 2001).

The MYB and bHLH transcription factors in rice have been well investigated. *OsC1*, R2R3-MYB-type transcription factor, is localized on rice chromosome 6 and it’s homologous to *ZmC1* (Reddy et al., 1998), a key anthocyanin biosynthesis gene in maize. It is also found that the *C1* gene is associated with the color of rice glume tip (Takahashi, 1957). *OsB1* and *OsB2*, the bHLH genes, both participate in anthocyanin regulation in rice but they are functionally redundant (Sakamoto et al., 2001). In order to function, both of them must interact with MYB-like transcription factors. Later, the *Kala4* bHLH gene is cloned and its functional analysis shows that *Kala4* can activate *EBGs* and *LBGs* to produce their respective specific pigments (Oikawa et al., 2015). Recently it’s reported that *OsTTG1*, WD40-type genes, is localized on rice chromosome 2 (Yang et al., 2021). Its protein is positioned in the nucleus and can physically interact with Kala4, OsC1, OsDFR, and Rc.

ZBXN 1 has a wealth of anthocyanins and total flavonoids. In this study, we constructed RILs with ZBXN 1 as the female plant and MH63 as the male plant by the method of single seed descent, measured and analyzed the content of anthocyanins and total flavonoids in different generations of this population by chemical analysis technique to study the characteristics and genetic properties between anthocyanins and total flavonoids, which can provide reference for the selection and breeding of functional rice enriched in anthocyanins and total flavonoids.

## 2 Materials and methods

### 2.1 Plant materials

The functional rice variety ZBXN 1 was bred by the Special Rice Research Institute of Yushan County, Jiangxi Province and selected from the parents of the August glutinous rice and the mutated purple glutinous rice. The process of selection and breeding was as follows: the local variety of August glutinous rice was crossed with the local variety of purple glutinous rice as the parent, and the F_1_ generation was backcrossed with the parent to obtain BC_1_F_1_. In 2006, the strain was approved by the Jiangxi Provincial Variety Approval Committee and named Ganwannuo6.

### 2.2 Population construction

The RILs used in this study consisted of 81 lines, which were obtained by crossing ZBXN 1 as the female plant and MH63 as the male plant to obtain the F_1_, and then backcrossing the F_1_ with MH63 to obtain BC_1_F_1_, and thereafter using the single seed descent method to obtain. In this experiment, three generations of BC_1_F_6_, BC_1_F_7_ and BC_1_F_8_ of RILs were finally selected as the study materials, noted as RIL6, RIL7 and RIL8, and all seeds were collected for the determination of anthocyanin and total flavonoid contents in brown rice.

### 2.3 Determination of anthocyanin content in brown rice

The experiment referred to the “Determination of anthocyanins in foods of plant origin by high performance liquid chromatography” (NY/T 2640-2014), and the content of anthocyanins in brown rice was determined by high performance liquid chromatography method. Accurately weighed 2g brown rice sample into 50ml colorimetric tube, added the prepared anthocyanin extraction solution and fixed the volume to the scale. Vortex shook for 1min and then used ultrasonic extraction for 1h. After extraction, put the colorimetric tube into boiling water bath and hydrolyzed for 1h, took it out and left it cool down, and then added the extraction solution to the scale in constant volume again. Subsequently, let it stand for a few minutes, took the supernatant and passed it through 0.45um aqueous filter membrane into the machine directly. In the chromatographic conditions, the mobile phase was adjusted to 1% acetonitrile formate solution and the gradient elution conditions were adjusted from a complex elution gradient to a simple gradient, which saved time and effort in the whole process on the basis of ensuring the accuracy of experimental data.

### 2.4 Determination of total flavonoid content in brown rice

In this experiment, referring to the industry standard “Determination of total flavonoids in buckwheat and its products” (NY/T 1295-2017), the pretreatment method was partially optimized, and the total flavonoid content in brown rice was determined by spectrophotometric method. Accurately weighed 2g brown rice sample and placed it in a 100 mL stoppered triangular flask, absorbed 30 mL of aqueous methanol solution, covered the stopper tightly, placed the triangular flask in a constant temperature water bath shaker at 65 °C and shook it at 160 r/min for 2 h. The filtrate was placed in a 50 mL volumetric flask. The filter paper and residue were washed with aqueous methanol solution. The filtrate was combined, cooled to room temperature, and then fixed the volume to the sacle with aqueous methanol solution. Meanwhile, a standard curve was made with 0.0500 mg·mL^-1^ of Rutin working solution. Absorbed 1 mL of the solution to be measured into a 10 mL volumetric flask, added 2 mL of 0.1 mol·L^-1^ Aluminium trichloride solution and 3 mL of 0.1 mol·L^-1^ potassium acetate solution according to the preparation method of Rutin working solution, and fixed the volume to the scale with methanol aqueous solution. Then shook it well, and left it at room temperature for 30 min, and centrifuged it at 4000 r·min^-1^ for 10 min. The absorbance value was measured the absorbance value A420 at 420 nm. The total flavonoid content was calculated by substituting it into the equation of standard curve.

## 3 Results

### 3.1 Endogenous analysis of anthocyanin between ZBXN 1 and MH63

**Figure 1:**
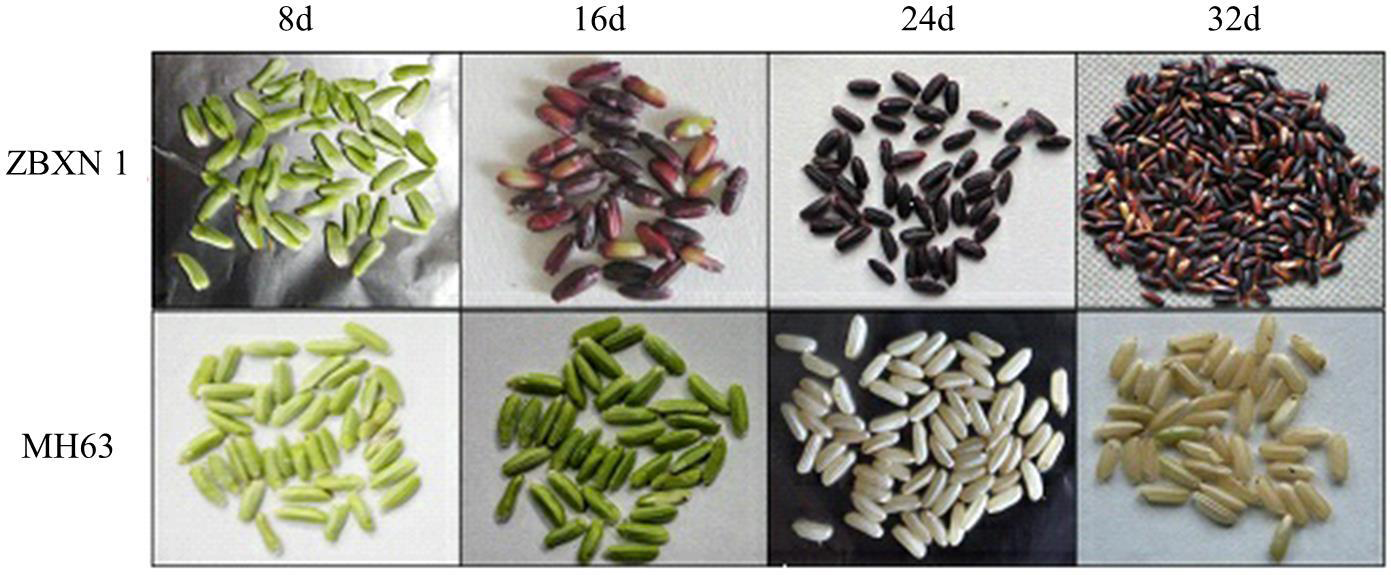
Grain color of ZBXN and MH63 at different filling stages. Grain color changes were observed between ZBXN 1 and MH63 at 8, 16, 24 and 32 days after flowering.

To investigate the anthocyanin development rule of parents ZBXN 1 and MH63, the experiment observed the phenotypes of parents’ grains at different periods (8, 16, 24 and 32 days after flowering) and measured their anthocyanin content. The color of the grains of ZBXN 1 gradually deepened as developing, with the darkest color of the seeds at 24 days after flowering and eventually fading to purple, however, MH63 did not have the color change of ZBXN 1 and it eventually showed the color of conventional rice (**Figure 1**).

**Figure 2:**
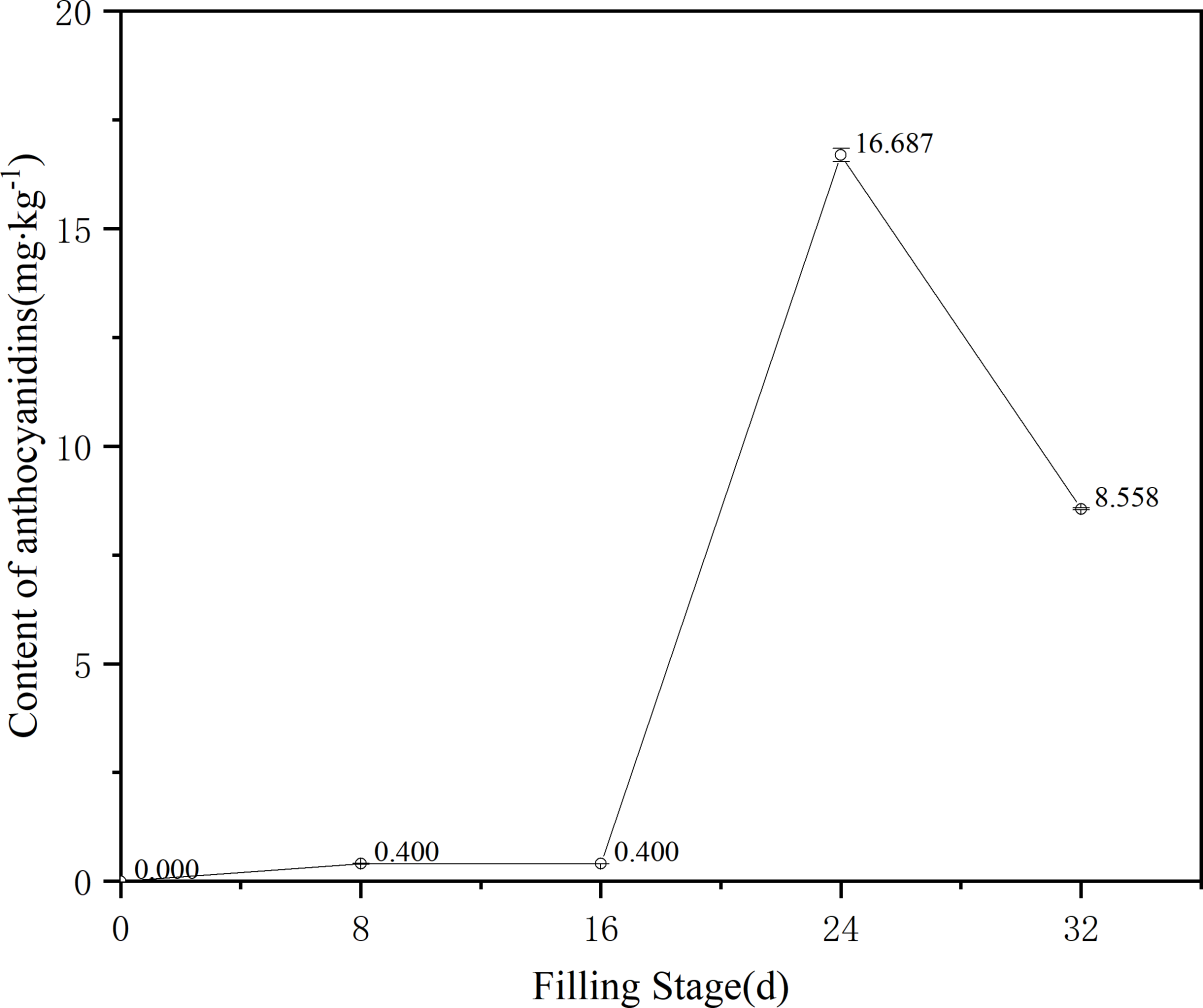
Change trend of anthocyanidins of different filling color in ZBXN 1. Determination of anthocyanin content of ZBXN 1 at 0, 8, 16, 24 and 32 days after flowering by HPLC.

The experiment further analyzed the anthocyanin content of ZBXN 1 at different filling stages by liquid chromatography, and the results showed an S-shaped curve variation (**Figure 2**). After flowering, the content of anthocyanins, from 0 to 16 days, showed a stable change and low content, at 8 d there are only peony pigments, while from 16 to 24 days anthocyanins showed a significant increase in content and the content was four times higher than that before 16 days. The content decreased slightly from 24 to 32 days at maturity. The anthocyanin content was half of that of 24 days. In summary, it can be seen that the anthocyanin synthesis of ZBXN 1 was significant during the developmental period from 16 to 24 days during the filling period, and the anthocyanin content decreased slightly when it reached the maturity period.

### 3.2 Endogenous analysis of total flavonoids between ZBXN 1 and MH63

**Figure 3:**
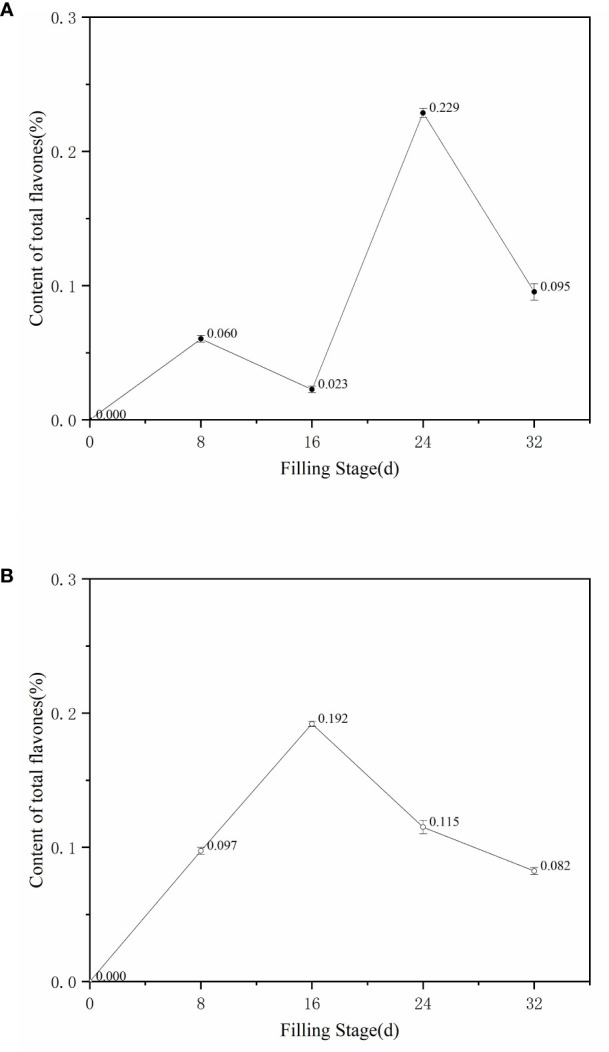
Change trends of total flavones content of different filling color in ZBXN 1 and MH63. The total flavonoid contents of ZBXN 1 **(A)** and MH63 **(B)** were measured at 0, 8, 16, 24 and 32 days after flowering.

The contents of ZBXN 1 and MH63 showed opposite S-shaped curve changes at different filling stages (**Figures 3A, B**), the total flavonoid content of ZBXN 1 decreased from 0.06% to 0.023% at 8-16 days after flowering, and increased sharply to a maximum of 0.229% from 16 to 24 days after flowering, but it decreased to 0.094% from 24 to 32 days after flowering. The total flavonoid content of MH63 increased from 0.095% to 0.192% from 8 to 16 days after flowering, and then decreased gradually to 0.115% from 16 to 32 days after flowering. Finally, the total flavonoid content at maturity was 0.082%.The total flavonoid contents of ZBXN 1 and MH63 were similar at the final maturity stage. Therefore, it can be seen that total flavonoids of ZBXN 1 and MH63 showed an inverse relationship from 0 to 24 days after flowering and were consistent from 24 to 32 days after flowering.

### 3.3 Anthocyanin and total flavonoid endogenous analysis of ZBXN 1

The trend graph of anthocyanin content results at different filling stages of ZBXN 1 was compared with its trend graph of total flavonoid content (**Figures 2**, **3A**). From the graph, we can see that anthocyanin and total flavonoid content showed mostly the same trend throughout the rice growth and development to maturity, and both showed an increasing trend at 8 d after the beginning of grouting, except that the total flavonoid content developed relatively faster than the anthocyanin. From 8 to 16 d, the total flavonoid was on a declining curve, but anthocyanin content tended to be stable. From 16 to 24 d, both contents showed sharp increases consistently, and from 24 to 32 d, they decreased to a stable state. This finding demonstrates that ZBXN 1 is a very high-quality rice variety that can be widely used as an intermediate material for breeding different functional special crops.

### 3.4 Anthocyanin and total flavonoid content of the RIL population

The RIL population was constructed by crossing ZBXN 1 as the female plant and MH63 as the male plant, and 81 long dominant materials were selected by measurement. In this study, the anthocyanin and total flavonoid contents of 81 RILs lines of different generations were determined by liquid chromatography.

**Figure 4:**
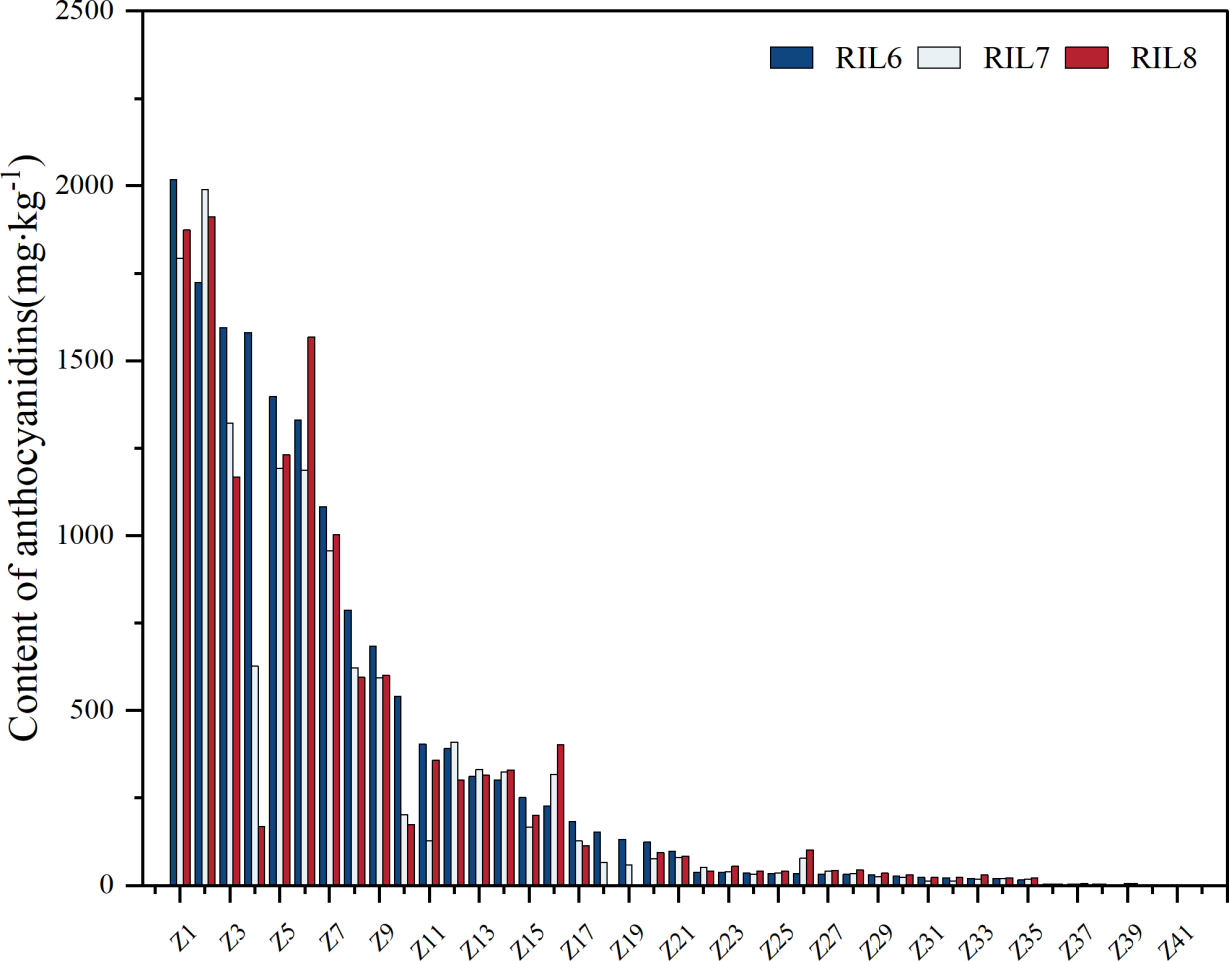
Anthocyanin content variation for different generations in RILs. The anthocyanin content of 81 lines of RILs samples from different generations was determined separately, and 41 samples were finally determined to contain anthocyanins by censoring out those samples with 0 mg/kg of detection. RIL6 represents BC_1_F_6_, RIL7 represents BC_1_F_7_, and RIL8 represents BC_1_F_8_.

Excluding the samples with 0 mg/kg of detection, the anthocyanin content of the 41 lines was finally determined and compared. The study of anthocyanin content in different generations of RIL populations showed that the overall analysis of anthocyanin genetic stability in different generations of the same RIL line was relatively stable (**Figure 4**). Among Z1, Z2, Z3, Z4 and Z6 with anthocyanin content over 1500 mg/kg, the content of anthocyanin in Z4 showed the largest change, and the content of anthocyanin decreased step by step. Z1 showed the smallest change, indicating that Z1 had better stability and higher overall content than Z4, while Z2 and Z3 were relatively better. Among Z5, Z7, Z8, Z9 and Z10 with anthocyanin content ranging from 500 mg/kg to 1500 mg/kg, Z10 had the biggest change in anthocyanin content, and also showed a decreasing trend, while other samples were relatively stable. The relative content of anthocyanin in Z11 to Z41 with 0 mg/kg~500 mg/kg was stable, and the anthocyanin content in Z13 was basically unchanged in three different generations, showing a super stable inheritance. Therefore, the anthocyanin content of 41 RILs samples was relatively stable at different generations. In addition, the total content of anthocyanins in RILs population was less than 500 mg/kg, accounting for 75.61%. The content of 500 mg/kg-1500 mg/kg and higher than 1500 mg/kg accounted for about 12.2%. In conclusion, the anthocyanin content of RILs offspring is generally low, but the content of individual family samples is high and has a stable inheritance. The average anthocyanin content of parent ZBXN 1 was 319.31 mg/kg, and that of parent MH63 was 0 mg/kg. Z1 had the highest anthocyanin content, with a mean value of 1894.75 mg/kg, which was not only significantly better than that of the parents, but also had good stability.

**Figure 5:**
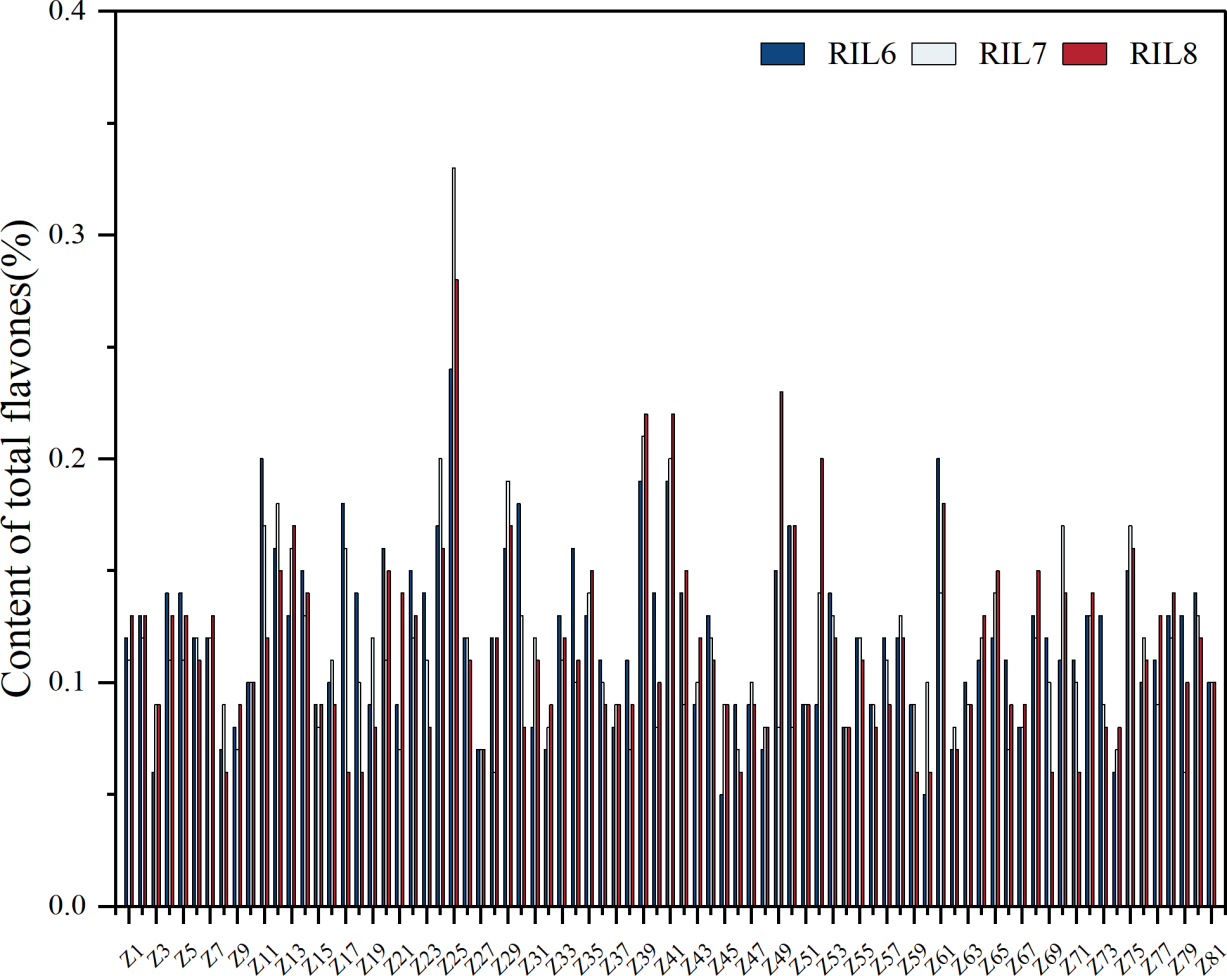
Total flavones content determination for different generations in RILs. The total flavonoid content was determined separately for 81 families of RILs samples of different generations. RIL6 represents BC_1_F_6_, RIL7 represents BC_1_F_7_, and RIL8 represents BC_1_F_8_.

It can be seen from the changes of total flavonoids content in different generations of each RILs sample that the overall total flavonoids are genetically stable (**Figure 5**). Z25, where the total flavonoids content is more than 0.3% and the difference between the highest and lowest flavonoids content is 0.09%, which can be ignored, has good stability. Among Z11, Z24, Z39, Z41, Z49, Z52, Z61 with 0.2%-0.3% total flavonoids content, Z49 showed the largest content change, and the difference between the highest and lowest content was 0.15%, indicating poor genetic stability. The variation of total flavonoids content in Z39 and Z41 was 0.03%, which showed good stability. Samples with the content of 0 ~0.2% were stable. The average content of total flavonoids of parent ZBXN 1 was 0.15% and the content of parent MH63 was 0.13%. But the content of total flavonoids of the highest content of Z25 was 0.33%, which indicated that Z25 was obviously better than that of the parent, and has good stability.

## 4 Discussion

**Figure 6:**
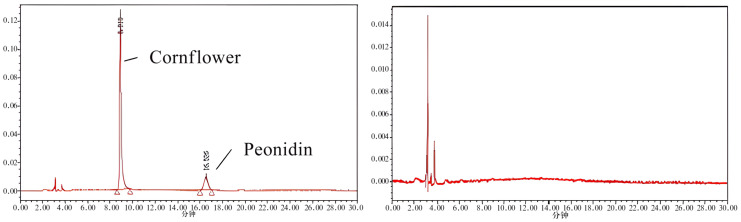
The HPLC chromatogram of ZBXN 1 and MH63. The chromatogram on the left is for ZBXN 1, and the one on the right is for MH63.

The genetic mechanism of seed coat pigmentation in colored rice has been studied since 1928, and there are many reports on the genetic mechanism of anthocyanins in rice thus far. But the conclusions obtained are not uniform because of the materials selected and the different criteria for classifying seed coat color (Causse et al., 1994; Yoshimura et al., 1997; Sweeney et al., 2006). In this experiment, the anthocyanin content of RIL populations of different generations showed that the overall analysis of anthocyanin genetic stability of the same RIL line in different generations was relatively stable. Among the RIL population grown for three generations, Z1 performed the best and could reach an average anthocyanin content of 1894.75 mg/kg, which was significantly better than the parents. Therefore, the anthocyanin content of ZBXN 1 can be improved through scientific research in the future, and the anthocyanin content of ZBXN 1 has some stability in combination with the results of anthocyanin analysis of the RIL population. According to the anthocyanin endogenous analysis of ZBXN 1 and MH63, the anthocyanin content of ZBXN 1 showed an S-shaped curve during the germination period, and the significant period of anthocyanin synthesis was from 16–24 days during the germination period, while MH63 did not contain the anthocyanin. According to the HPLC chromatogram (**Figure 6**), it was obvious that the brown rice contained only two pigments, cornflower and Peonidin. The retention time of the cornflower peak was 8.873 min and 16.21 min for Peonidin, while the peak of MH63 didn’t appear at the corresponding location.

It was observed from the study of three generations of RIL populations that the difference in total flavonoid content in the population was basically negligible, so the change in total flavonoid content corresponding to different generations of RIL populations could indicate that the total flavonoid content was stable. The highest total flavonoid content of Z25 was 0.33%, which was remarkably better than that of the parents, while the total flavonoid content between parents was 0.15% for ZBXN 1 and 0.13% for MH63, which meant there was a low correlation between total flavonoid content and environment and geographical area. The endogenous analysis of total flavonoids between ZBXN 1 and MH63 showed the opposite trend, but at last the total flavonoid contents of ZBXN 1 and MH63 converged at maturity. Comparing anthocyanins and total endogenous flavonoids in ZBXN 1 alone, they basically showed the same trend, except for a slight decrease in total flavonoids at 8–16 days of germination.

There are only a few studies on the brown rice flavonoid content of different rice backcross progeny. One researcher analyzed the total flavonoid content of brown rice from backcross progeny of core germplasm in Yunnan and screened the highest content of 1.268% ± 0.05% in japonica rice progeny obtained by the crossing of White Liangdaogu and Hexi353 (Jin et al., 2010). According to Ling, it was found that colored rice is easier to screen rice seed resources with high flavonoid content, and the darker the seed coat of the rice is, the higher the flavonoid content, which indicated that dark colored rice possesses rich antioxidant properties (Sun et al., 2002). In summary, the lines with high anthocyanin content detected in this experiment can be used as breeding intermediate materials for the selection of new high-quality varieties rich in anthocyanins.

## Data availability statement

The original contributions presented in the study are included in the article/**Supplementary Material**. Further inquiries can be directed to the corresponding authors.

## Author contributions

XZ and WX performed all the experiments. WX, HH, and DZ designed the research. HJ helped collect samples, JF, ML, JB, JX, YC provided suggestions for experiments and manuscripts. All authors have read and approved the content of the final manuscript.
