# Supplementary material for: Analysis of anthocyanins and total flavonoids content in functional rice and its recombination inbred lines
Source: Front Plant Sci. 2023 Mar 15;14:1113618. doi: 10.3389/fpls.2023.1113618 (PMC10050882; doi:10.3389/fpls.2023.1113618)
Supplement: Supplementary file 1 [file DataSheet_1.pdf]

Supplementary Material

Article Title

Xinyi Zhou[1†], Wei Xie[1†], Hao Jing[1], Junru Fu[1], Maomao Li[2], Jianmin Bian[1], Jie Xu[1], Yicong Cai[1], Haohua He[1]*, Dahu Zhou[1]*

*** Correspondence:** Corresponding Author: email@uni.edu

Corresponding Author Haohua He[1]*, Dahu Zhou[1]*

TABLE S1. Anthocyanin content in RIL populations of three generations. Z13 is ZBXN 1, Z42 is MH63 and the unit of measurement is mg·kg^{-1}.

No.	RIL6	RIL7	RIL8
Z1	2016.77	1793.89	1873.60
Z2	1722.79	1990.50	1912.00
Z3	1595.15	1321.37	1167.79
Z4	1580.14	627.00	168.57
Z5	1397.08	1192.39	1230.60
Z6	1331.62	1186.16	1567.74
Z7	1083.10	956.55	1003.50
Z8	787.29	622.34	595.67
Z9	683.73	593.73	600.73
Z10	541.10	202.22	174.65
Z11	404.70	128.64	357.84
Z12	390.69	410.14	300.93
Z13	312.02	330.60	315.30
Z14	301.99	325.52	330.10
Z15	250.94	166.50	200.94
Z16	227.60	317.80	402.60
Z17	183.06	127.06	114.54
Z18	153.43	65.26	0.00
Z19	132.53	57.80	2.69
Z20	123.95	75.89	94.85
Z21	98.33	78.86	83.60
Z22	38.43	51.44	41.44
Z23	37.58	39.61	54.48
Z24	35.60	31.63	40.40
Z25	34.35	35.60	41.30
Z26	33.10	77.60	102.03
Z27	32.11	40.60	42.10
Z28	32.03	33.10	44.20
Z29	30.20	25.77	35.36
Z30	27.04	23.60	31.40
Z31	22.40	13.03	23.60
Z32	21.84	12.41	22.30
Z33	20.30	17.84	30.10
Z34	19.70	20.47	21.60
Z35	15.88	17.44	21.60
Z36	4.31	3.21	4.05
Z37	4.11	3.22	6.36
Z38	3.60	3.56	2.49
Z39	3.06	5.06	5.63
Z40	2.21	3.12	2.63
Z41	1.60	3.10	2.96
Z42	0.00	0.00	0.00

TABLE S2. Anthocyanin content in RIL populations of three generations. Z13 is ZBXN 1, Z42 is MH63 and the unit of measurement is a percentage (%).

No.	RIL6	RIL7	RIL8
Z1	0.12	0.11	0.13
Z2	0.13	0.12	0.13
Z3	0.06	0.09	0.09
Z4	0.14	0.11	0.13
Z5	0.14	0.11	0.13
Z6	0.12	0.12	0.11
Z7	0.12	0.12	0.13
Z8	0.07	0.09	0.06
Z9	0.08	0.07	0.09
Z10	0.10	0.10	0.10
Z11	0.20	0.17	0.12
Z12	0.16	0.18	0.15
Z13	0.13	0.16	0.17
Z14	0.15	0.13	0.14
Z15	0.09	0.08	0.09
Z16	0.10	0.11	0.09
Z17	0.18	0.16	0.06
Z18	0.14	0.10	0.06
Z19	0.09	0.12	0.08
Z20	0.16	0.11	0.15
Z21	0.09	0.07	0.14
Z22	0.15	0.12	0.13
Z23	0.14	0.11	0.08
Z24	0.17	0.20	0.16
Z25	0.24	0.33	0.28
Z26	0.12	0.12	0.11
Z27	0.07	0.07	0.07
Z28	0.12	0.06	0.12
Z29	0.16	0.19	0.17
Z30	0.18	0.13	0.08
Z31	0.08	0.12	0.11
Z32	0.07	0.08	0.09
Z33	0.13	0.11	0.12
Z34	0.16	0.10	0.11
Z35	0.13	0.14	0.15
Z36	0.11	0.10	0.09
Z37	0.08	0.09	0.09
Z38	0.11	0.07	0.09
Z39	0.19	0.21	0.22
Z40	0.14	0.08	0.10
Z41	0.19	0.20	0.22
Z42	0.14	0.09	0.15
Z43	0.09	0.10	0.12
Z44	0.13	0.12	0.11
Z45	0.05	0.09	0.09

No.	RIL6	RIL7	RIL8
Z46	0.09	0.07	0.06
Z47	0.09	0.10	0.09
Z48	0.07	0.08	0.08
Z49	0.15	0.08	0.23
Z50	0.17	0.08	0.17
Z51	0.09	0.09	0.09
Z52	0.09	0.14	0.20
Z53	0.14	0.13	0.12
Z54	0.08	0.08	0.08
Z55	0.12	0.12	0.11
Z56	0.09	0.09	0.08
Z57	0.12	0.11	0.09
Z58	0.12	0.13	0.12
Z59	0.09	0.09	0.06
Z60	0.05	0.10	0.06
Z61	0.20	0.14	0.18
Z62	0.07	0.08	0.07
Z63	0.10	0.09	0.09
Z64	0.11	0.12	0.13
Z65	0.12	0.14	0.15
Z66	0.11	0.07	0.09
Z67	0.08	0.08	0.09
Z68	0.13	0.12	0.15
Z69	0.12	0.10	0.06
Z70	0.11	0.17	0.14
Z71	0.11	0.10	0.06
Z72	0.13	0.13	0.14
Z73	0.13	0.09	0.08
Z74	0.06	0.07	0.08
Z75	0.15	0.17	0.16
Z76	0.10	0.12	0.11
Z77	0.11	0.09	0.13
Z78	0.13	0.12	0.14
Z79	0.13	0.06	0.10
Z80	0.14	0.13	0.12
Z81	0.10	0.10	0.10
